# Antithrombotic Treatment in Lower Extremity Peripheral Arterial Disease

**DOI:** 10.3389/fcvm.2021.773214

**Published:** 2021-12-23

**Authors:** Anders Gottsäter

**Affiliations:** Department of Internal Medicine, Skåne University Hospital, University of Lund, Malmö, Sweden

**Keywords:** atherosclerosis, antiplatelet treatment, anticoagulation, peripheral atherosclerosis, PAD

## Abstract

Lower extremity arteries might be affected by atherosclerotic peripheral arterial disease (PAD), or by embolization causing ischaemic symptoms. Patients with PAD often have widespread atherosclerosis, and progression of PAD is associated with increased risk for both other cardiovascular events and cardiovascular mortality. Peripheral arterial disease patients should therefore be offered both non-pharmacological and pharmacological secondary prevention to reduce the risk for future ischemic arterial complications. This review is focussed on the rationale for recommendations on antiplatelet and anticoagulant treatment in PAD. Asymptomatic PAD does not warrant either anticoagulant or antiplatelet treatment, whereas patients with ischaemic lower extremity symptoms such as intermittent claudication or critical limb ischemia caused by atherosclerosis should be offered platelet antiaggregation with either low dose aspirin or clopidogrel. Combined treatment with aspirin and low-dose of the direct oral anticoagulant (DOAC) rivaroxaban should be considered and weighed against bleeding risk in symptomatic PAD patients considered at high risk for recurrent ischaemic events and in patients having undergone endovascular or open surgical intervention for PAD. Patiens with cardiogenic embolization to lower extremity arteries should be recommended anticoagulant treatment with either one of the DOACs (apixaban, dabigatran, edoxaban, and rivaroxaban) or warfarin.

## Introduction, Background

Peripheral arterial disease (PAD) is a common atherosclerotic manifestation (1, 2) most often occurring in lower extremity arteries. The condition might be asymptomatic, but both focal atherosclerotic lesions in the peripheral arteries and cardiogenic embolization to the lower extremities might cause ischaemic symptoms such as intermittent claudication defined as pain induced by walking (3, 4), or acute or chronic limb threatening ischemia (CLTI) defined as rest pain or ulceration (3, 4).

Patients with atherosclerotic PAD have widespread atherosclerosis and higher rates of cardiovascular events than patients with cardio- or cerebrovascular diseae (5). As both a low ankle-brachial index (ABI) (6) and progression of PAD (7) are related to increased risk for cardiovascular events and mortality, efficient treatment of atherosclerotic risk factors is recommended in PAD patients (3, 4).

Thrombolytic, endovascular, and open surgical treatment in the acute or chronic stages of PAD caused by peripheral atherosclerosis or embolization are covered in current guidelines (3, 4) together with recommendations on smoking cessation (8), lipid (9), and blood pressure (10) lowering. This review is focussed upon antithrombotic treatment as secondary prevention of cardiovascular mortality and morbidity in patients with lower extremity ischemia caused by either peripheral atherosclerosis or cardiac embolization (Tables 1, 2).

**Table 1:** Summary of recommendations and concerns on antithrombotic treatment in peripheral arterial disease (PAD).

	**Asymptomatic PAD without other symptomatic atherosclerosis**	**Stable symptomatic PAD**	**After endovascular intervention for PAD**	**After open surgery for PAD**	**Peripheral ischemia caused by cardioembolic disease**
First line	No antithrombotic therapy	ASA or clopidogrel	ASA and low dose rivaroxaban	ASA or clopidogrel	DOAC
Alternative		ASA and low dose rivaroxaban	ASA and clopidogrel	ASA and low dose rivaroxaban	VKA
Alternative			ASA or clopidogrel	VKA if venous bypass	
Concerns		Evaluate bleeding risk	Evaluate bleeding risk Uncertainty on duration of combination	Evaluate bleeding risk	
References	(3, 4, 11, 12)	(3, 4, 13–19)	(3, 4, 20–23)	(3, 4, 19, 23–25)	(3, 4, 26–33)

*ASA, aspirin; DOAC, direct oral anticoagulant; VKA, vitamin K antagonist*.

**Table 2:** Studies of antithrombotic therapy in peripheral arterial disease (PAD).

**Study (first author or eponym, year)**	**References**	**Patient population**	**Comparison**	**Follow up** **(mean or median, months)**	**Number of patients**	**RR or HR primary outcome (95% CI)**	**RR or HR major bleeding** **(95% CI)**
Fowkes et al., 2010	(11)	Asymptomatic PAD	Aspirin or placebo	98	28,980	1.03 (0.84–1.27)	1.71 (0.99–2.97)
Belch et al., 2008	(12)	Asymptomatic PAD with diabetes	Aspirin or placebo	80	1,276	0.98 (0.76–1.26)	
CAPRIE, 1996	(14)	Prior PAD, stroke, or MI	Aspirin or clopidogrel	23	19,185	Relative risk reduction (%) 8.7 (0.3–16.5)	NS for ICH, *p* = 0.23
Hiatt et al., 2017	(15)	Symptomatic PAD	Ticagrelor or clopidigrel	30	13,885	1.02 (0.92–1.13)	1.10 (0.84–1.43)
Bonaca et al., 2016	(34)	PAD and MI	Ticagrelor and aspirin or aspirin only	36	1,143	Absolute risk reduction (%) 4.1 (−1.07–9.29)	1.32 (0.41–4.29)
Bhatt et al., 2006	(35)	Cardiovascular disease or multiple risk factors	Clopidogrel and aspirin or aspirin only	28	15,603	0.93 (0.83–1.05)	1.25 (0.97–1.61)
Anand et al., 2007	(36)	PAD	Aspirin and warfarin or aspirin only	35	2,161	0.92 (0.73–1.16)	3.41 (1.84–6.35)
Anand et al., 2018	(18)	Stable lower exrtremity or carotid PAD	Low dose rivaroxaban and aspirin or aspirin only	21	7,470	0.72 (0.57–0.90)	1.61 (1.12–2.31)
Tepe et al. 2012, Strobel et al. 2013	(21, 22)	After endovascular PAD intervention	Clopidogrel and aspirin or aspirin only	12	80	NS for revascularization, *p* = 0.35	
Bonaca et al. 2020, Hiatt et al. 2020	(23, 37)	After PAD intervention	Low dose rivaroxaban and aspirin or aspirin only	36	6,564	0.85 (0.76–0.96)	1.43 (0.97–2.10)
Dutch BOA, 2000	(38)	After open surgical PAD intervention	Oral anticoagulant or aspirin	21	2,690	0.95 (0.82–1.11)	1.96 (1.42–2.71)
Belch et al., 2010	(25)	After open surgical PAD intervention	Aspirin and clopidogrel or aspirin only	12	851	0.98 (0.78–1.23)	NS, 2.1 vs. 1.2%
Johnson et al., 2002	(39)	After open surgical PAD intervention	Oral anticoagulant and aspirin or aspirin only	Up to 60	831	NS for patency in whole group	1.41 (1.09–1.84) for death

*CI, confidence interval; HR, hazard ratio; ICH, intracranial hemorrhage; MI, myocardial infarction; NS, no significance; RR, risk ratio. As modes of reporting, primary endpoints, and definitions of major bleeding differ in the different studies, please see the original publications for details*.

## Antithrombotic Treatment in Asymptomatic Peripheral Atherosclerotic Disease

No beneficial effects of antithrombotic treatment have been established in patients with asymptomatic PAD, i.e., a low ABI without symptoms from the lower extremities or other concomitant vascular disease. In 3,350 asymptomatic subjects from the general population with ABI ≤ 0.95 detected at screening, aspirin did not confer any significant reduction in vascular events compared with placebo (11). Neither could any benefits with regard to cardiovascular events or major amputation be shown when effects of aspirin 100 mg daily were compared to placebo in patients with asymptomatic PAD (ABI ≤ 0.99) and concomitant diabetes (12). Current guidelines (3, 4) do therefore not recommend antiplatelet treatment in PAD patients without other symptomatic manifestations of atherosclerotic disease (Table 1).

## Antithrombotic Treatment in Symptomatic Stable Peripheral Atherosclerotic Disease

The antiplatelet trialists'meta-analysis (13) published already in 2002 established that different types of antiplatelet therapy reduce the risk of vascular death, myocardial infarction, and stroke by approximately 25% among patients with mainly symptomatic coronary and cerebrovascular disease. Patients with different manifestations of PAD were included as a subgroup in the meta-analysis, and a 23% odds reduction for vascular events could be demonstrated in this group (13). Randomized placebo-controlled studies performed exclusively in patients with stable PAD showing benefits of low dose aspirin for reduction of symptoms or cardiovascular events are lacking, however, whereas the ADP-receptor blocker thienopyridine ticlopidine was shown to be beneficial in this regard already in 1990 in a small study of 687 patients (40). The use of ticlopidine is limited by its gastroenterological and hematological side effects, however. Another thienopyridine, clopidogrel, was therefore compared with aspirin in patients with either myocardial infarction, ischemic stroke, or PAD in the CAPRIE trial (14). In the CAPRIE subgroup of 6,452 patients with PAD, clopidogrel reduced both cardiovascular mortality [hazard ratio (HR) 0.76, 95% confidence interval (CI) 0.64–0.91] and major cardiovascular adverse events (HR 0.78, 95% CI 0.65–0.93) compared to aspirin (14). When clopidogrel was later compared with ticagrelor in the EUCLID trial (15) conducted exclusively among symptomatic PAD patients, no significant differences between these two compounds could be demonstrated either regarding cardiovascular events or bleeding complications (15).

The protease-activated receptor 1 antagonist vorapaxar was found to reduce the risk of acute limb ischemia in the PAD subgroup in the TRA2°P-TIMI 50 study (41), but is also associated with increased risk for intracranial hemorrhage in patients with prior ischaemic cerebrovascular disease (34). In a meta-analysis of 49 RCTs comprising 34,518 patients neither aspirin, ticlopidine, ticagrelor, cilostazol, picotamide, or vorapaxar in monotherapy was superior to clopidogrel regarding the combined endpoint of efficacy and safety in PAD patients (16).

As no placebo arm was included in CAPRIE (14) and as EUCLID (15) lacked an aspirin arm, however, the evidence for platelet antiaggregation in PAD can still be somewhat disputed. Current guidelines (3, 4) recommend long-term single antiplatelet treatment with either aspirin or clopidogrel in symptomatic stable PAD patients who are not candidates for anticoagulant treatment as outlined below, provided they have no contra-indications such as increased bleeding risk, prior side effects of pharmacologic treatment, cognitive dysfunction, or other disabilities.

### Dual Antiplatelet Therapy

Dual antiplatelet therapy (DAPT) with a combination of 75–162 mg of aspirin and 75 mg of clopidogrel was evaluated in the CHARISMA trial (35) performed in 15,603 patients with either established vascular disease or multiple risk factors for atherosclerosis. Dual antiplatelet therapy compared to aspirin alone conferred no significant risk reduction (RR) for the primary study endpoint of either cardiovascular death, myocardial infarction, or stroke (RR 0.93; 95% CI 0.83–1.05; *p* = 0.22), whereas a significant RR was demonstrated for the secondary endpoint; hospitalization for ischemia or revascularization (RR 0.92; 95% CI 0.86–0.995; *p* = 0.04). In the subgroup of 3,096 CHARISMA patients with PAD (17) of which the vast majority were symptomatic, however, both rates of myocardial infarction (2.3 vs. 3.7%; *p* = 0.029), and hospitalization for ischemic events (16.5 vs. 20.1%; *p* = 0.011) were lower with DAPT than with aspirin alone. Rates of severe, fatal, or moderate bleeding did not differ, but minor bleeding occurred more often with DAPT (34.4 vs. 20.8%; *p* = 0.001). As a subgroup analysis of a negative trial should not be used as a basis for treatment decisions, there is no guideline support for routine use of DAPT in patients with stable PAD (3, 4). This conclusion is also supported by results from the above mentioned meta-analysis of 49 RCTs comprising 34,518 patients (16).

### Combined Antiplatelet and Anticoagulant Therapy

When the combination of antiplatelet treatment and full dose anticoagulation with warfarin was evaluated after myocardial infarction (42) it was found to be beneficial regarding risk for death, reinfarction or stroke, whereas no such benefits of combination therapy could be established when studying effects of the same combination in PAD patients in the WAVE trial (36). Furthermore, combination therapy also conferred unacceptable inceased bleeding rates in both study settings (36, 42).

When later evaluating the combination of aspirin with a direct oral anticoagulant (DOAC) in PAD patients, a lower dose of anticoagulation was therefore employed. The COMPASS study (18, 43) compared three different active treatments; a combination of low dose rivaroxaban 2.5 mg twice daily and aspirin 100 mg daily, rivaroxaban 5 mg twice daily, and aspirin 100 mg daily with corresponding placebos in 24,824 patients with stable coronary artery disease or PAD. In a subgroup analysis (18) of the 7,470 COMPASS patients with either stable lower extremity PAD or carotid artery disease, the combination of rivaroxaban 2.5 mg twice daily and aspirin 100 mg daily reduced both the primary composite endpoint cardiovascular death, myocardial infarction, or stroke (5 vs. 7%; *p* = 0.0047), and the primary PAD-related endpoint “major adverse limb events” including amputation (1 vs. 2%; *p* = 0.0037) compared to aspirin alone, whereas rivaroxaban 5 mg twice daily did not confer any definitive benefits compared to aspirin (18). The combination of rivaroxaban and aspirin combination also increased major bleeding compared with aspirin alone (3 vs. 2%; HR 1.61, 95% CI 1.12–2.31; *p* = 0.0089), however, mainly due to an increased risk for gastrointestinal bleeding.

European PAD guidelines (4) issued after the publication of COMPASS (18, 43) therefore recommend that a combination of ASA 100 mg daily and rivaroxaban 2.5 mg twice daily should be considered in stable PAD patients without high bleeding risk or other relevant contraindications. The same consideration is also recommended in the global guidelines for treatment of patients with the most serious form of PAD, CTLI (19).

## Antithrombotic Treatment After Intervention for Peripheral Atherosclerotic Disease

### Endovascular Intervention

Endovascular percutaneous transluminal angioplasty (PTA) with or without stent placement might increase the risk for thromboembolic events both by disrupting the endothelium or atherosclerotic plaques and by introduction of foreign material in the artery. This might activate platelets and coagulation factors, initiate atherothrombosis, and consequently increase the risk of arterial occlusion. In a systematic follow-up of nationwide Swedish registry data (44), the risk of non-fatal MI, ischemic stroke, or cardiovascular death 36 months after peripheral revascularization was 14% among patients with IC and and 34% among those with CLTI. Furthermore, the TRA2°P study (41, 45) confirmed that peripheral revascularization increased the risk of acute limb ischemia and the need for both urgent and elective reintervention. Particular interest has therfore been focussed on this patient group when assessing effects of antithrombotic treatment.

Antiplatelet therapy after endovascular revascularization of peripheral arteries has often been based on recommendations (46) based on studies of patients undergoing percutaneous coronary interventions (PCI), and many vascular units routinely recommend a combination of aspirin and clopidogrel for 1–3 months after peripheral revascularization. One month of DAPT is also recommended in the current version of the PAD guidelines issued by the European Society of Vascular Surgery and European Society of Cardiology (3). A thourough meta-analysis (20) of 5,464 publications in the field in 2016 revealed, however, that only one of the evaluated articles was relevant. In the MIRROR trial (21, 22) the combination of aspirin and clopidogrel was compared with monotherapy with aspirin after percutaneous angioplasty with or without stenting in the femoropopliteal segment. The 6-month results of MIRROR (21) were promising with lower need for target lesion revascularization with combination therapy, but after 12 months of follow-up (22), this difference was no longer detectable.

Authors of the meta-analysis concluded that the lacking evidence for DAPT after lower limb endovascular revascularization might partly be explained by the fact that interventionalists have already adopted the DAPT regime used after PCI (46), making it difficult to conduct new randomized trials of DAPT after endovascular revascularization in PAD (20).

Furthermore, in the recently published VOYAGER study (23) rivaroxaban 2.5 mg twice daily combined with aspirin 100 mg daily was compared to aspirin 100 mg and placebo in 6,564 patients revascularized due to symptomatic PAD. Revascularization had been performed with endovascular or hybrid methods in 65% of cases, and with open surgery in the remaining 35%. The majority of patients were treated because of intermitent claudication, but 23% had CLTI. The combined primary efficacy endpoint of cardiovascular death, myocardial infarction, stroke, acute limb ischemia, or amputation above ankle occurred in 17.3% and 19.9% of patients in the combination and aspirin only group, respectively (HR 0.85; 95% CI 0.76–0.96; *p* = 0.009) during 36 months, corresponding to an absolute RR of 2.6% and a number needed to treat (NNT) of 39. As the primary safety endpoint, major bleeding defined in accordance with the Thrombolysis In Myocardial Infarction (TIMI) classification (47), did not differ significantly between groups (2.7 and 1.9%, *p* = 0.07), and as the safety of rivaroxaban was later shown to be consistent regardless of concomitant clopidogrel use (37), it must be concluded that the evidence is far more solid for the use of the combination of low doses of aspirin and rivaroxaban after endovascular peripheral revascularization than for DAPT.

### Open Vascular Surgery

Full dose vitamin K antagonists was compared to aspirin in 2,690 patients having undergone infrainguinal bypass surgery in the Dutch Bypass Oral Anticoagulants or Aspirin (BOA) trial (38). The study was neutral (HR 0.95; 95% CI 0.82–1.11), but subgroup analyses revealed that vitamin K antagonism conferred a reduction in graft occlusion (HR 0.69; 95% CI 0.54–0.88) in patients receiving vein grafts, but an increased risk in those receiving prosthetic grafts (HR 1.26; 95% CI 1.03–1.55). The evidence for vitamin K antagonist use after venous bypass has later been considered as insufficient in a Cochrane analysis (24), however.

The CASPAR study (25) showed no additive effect of combining aspirin with clopidogrel after open bypass surgery in lower limb arteries regarding the composite primary efficacy endpoint of index-graft occlusion, revascularization, above-ankle amputation of the affected limb, or death, except for in the subgroup of patients with prosthethic grafts. The combination of aspirin and full dose warfarin after lower extremity bypass was associated with both increased morbidity and mortality (39).

Guidelines (3, 4, 19) therefore recommend single antiplatelet therapy after open surgery for PAD, although the different European guidelines mentions vitamin-K antagonists after venous bypass either as an alternative (4) or as an option for which evidence is weak and bleeding risk is higher compared to antiplatelet drugs (3) (Table 1).

As beneficial effects of combination therapy could be demonstrated also in the subgroup of VOYAGER patients having undergone revascularization with open surgical methods (23), however, the combination of low dose aspirin and rivaroxaban could well be considered also in this situation in patients without high bleeding risk or other contraindications.

## Anticoagulation in Patients With Peripherial ischemia Caused by Cardiac Embolization

Cerebral embolism is by far the most common and feared embolic consequences of atrial fibrillation (AF), and 80% of deaths related to cardiogenic embolism are caused by ischemic stroke (48). Atrial fibrillation is also the most common cause of peripheral embolism, however, and estimated to be present in 60–95% of patients undergoing surgery for acute limb ischemia (49). The yearly incidence of aortoiliac and lower-extremity arterial thromboembolism in AF is about 0.4%, corresponding to an excess risk of 4.0 (95% CI 3.5–4.6) in men and 5.7 (95% CI 5.1–6.3) in women (50).

Current European guidelines for AF (26) recommend assessment of the risk for systemic cardiac embolisation by evaluation of the factors below summarized in the CHA_2_DS_2_-VASc score (50). As anticoagulant treatment is recommended already in patients with CHA_2_DS_2_-VASc score ≥1 in men and ≥2 in women (26), and as a previous episode of thromboembolism (S) confers two points, all patients with permanent or paroxysmal AF who have suffered an episode of lower extremity embolism have a score of 2 or higher. After endovascular, open surgical, or thrombolytic treatment of the acute event, they should therefore be offered secondary prevention by full dose anticoagulation in the abscence of important contraindications. This recommendation also applies to patients with peripheral embolization caused by prosthethic heart valves or other cardiac sources of embolism (3).

As the presence of atherosclerotic peripheral vascular disease in itself confers one CHA_2_DS_2_-VASc point (26), most PAD patients with concomitant AF will qualify for anticoagulation also in the abscence of documented thromboembolic episodes in the lower extremities.

In patients with an established indication for anticoagulation undergoing endovascular PAD recanalization, European guidelines recommend consideration of a 1–12 month course of aspirin or clopidogrel as addition to the anticoagulant in the abscence of high bleeding risk (3, 4). After open surgical procedures for PAD in this patient group, on the other hand, only continued anticoagulation is recommended (3, 4).

Direct oral anticoagulant, the thrombin inhibitor dabigatran (27) or one of the factor Xa-inhibitors rivaroxaban (28), edoxaban (29), and apixaban (30) are first hand alternatives for anticoagulation. Meta-analysis (31) has established that treatment with these agents in comparison to warfarin confers 19% RR for stroke or other systemic embolism and a 51% RR in haemorrhagic stroke. Direct oral anticoagulant treatment was also associated with a non-significant 14% reduction in major bleeding risk, a 52% reduction in intracranial hemorrhage, and a 25% increase in gastrointestinal bleeding compared to warfarin (31). Although focus in the above trials (27–30) and meta-analysis (31) has been on stroke prevention with DOAC, a systematic literature review (32) confirmed that DOAC are also at least as effective as warfarin to reduce the risk for limb ischemia in patients with AF. Furthermore, among patients with AF and concomitant CLTI, the superiority of DOAC in comparison to either warfarin or antiplatelet therapy has been established in a retrospective cohort analysis (33).

Warfarin can of course still be used as an alternative for prevention of systemic thromboembolic events in patients with AF or other sources of embolism to peripheral arteries (26), however, and is superior to dabigatran in patients with mechanical heart valves (51) and to rivaroxaban in those with antiphospholipid syndrome (52). The therapeutic target is an international normalized ratio (INR) of 2.0–3.0. There is no evidence for warfarin treatment with lower INR-targets, or for combination treatment with warfarin in combination with aspirin or other antiplatelet agents in PAD.

## Emerging Role, Unmet Needs, and Gray Areas

The benefits of combined treatment with low doses of aspirin and rivaroxaban has been established in both stable PAD (18, 43) and after peripheral revascularization (23, 37), and this benefit increases with baseline risk in the patient (53). Bleeding risk with this treatment also has to be taken into account, however, and PAD patients with a perceived high risk for bleeding complications were excluded from the studies. We must therefore more clearly define the groups of PAD patients in which combination treatment with low doses of aspirin and rivaroxaban is safe and cost-effective in clinical practice.

Furthermore, we lack studies establishing the efficacy and safety of combined antitplatelet and full dose anticoagulant treatment after peripheral revascularization in patients with AF or other established indications for anticoagulation. To which patients in this group should platelet inhibition be added to the oral anticoagulation, and for how long after the intervention?

Neither do we know if PAD progression in itself, measured for example as a worsening ABI, is enough to warrant modification of antithrombotic therapy.

## Summary

Whereas asymptomatic PAD does not warrant either anticoagulant or antiplatelet treatment, patients with ischaemic symptoms such as intermittent claudication or CLTI caused by atherosclerosis should be offered platelet antiaggregation with either low dose aspirin or clopidogrel, and those with cardioembolic disease should be recommended full dose anticoagulant treatment with either DOAC or warfarin. Combined treatment with aspirin and low dose rivaroxaban should be considered and weighed against bleeding risk in symptomatic PAD patients with high risk for recurrent ischaemic events and in those having undergone peripheral endovascular or open surgical intervention. These concerns and recommendations are summarized in Table 1.

## Author's Note

AG is the only author of this review submitted for the research topic—Anticoagulation in cardiovascular diseases: evolving role, unmet needs, and gray areas.

## Author Contributions

The author confirms being the sole contributor of this work and has approved it for publication.

## Conflict of Interest

The author declares that the research was conducted in the absence of any commercial or financial relationships that could be construed as a potential conflict of interest.

## Publisher's Note

All claims expressed in this article are solely those of the authors and do not necessarily represent those of their affiliated organizations, or those of the publisher, the editors and the reviewers. Any product that may be evaluated in this article, or claim that may be made by its manufacturer, is not guaranteed or endorsed by the publisher.
